# Hyaluronic acid‐*g*‐lipoic acid granular gel for promoting diabetic wound healing

**DOI:** 10.1002/btm2.10402

**Published:** 2022-09-14

**Authors:** Shixi Zhang, Yuqing Pan, Zhiyuan Mao, Jiahui Zhang, Kunxi Zhang, Jingbo Yin, Chen Wang

**Affiliations:** ^1^ Department of Plastic and Reconstructive Surgery, Shanghai 9th People's Hospital Shanghai Jiao Tong University School of Medicine Shanghai People's Republic of China; ^2^ Department of Polymer Materials, School of Materials Science and Engineering Shanghai University Shanghai People's Republic of China

**Keywords:** antibacterial, diabetic wound healing, granular gel, lipoic acid, reactive oxygen species

## Abstract

Diabetic patients are prone to developing chronic inflammation after trauma and have persistent nonhealing wounds. Reactive oxygen species (ROS) and recurrent bacterial infections at the site of long‐term wounds also further delay skin wound healing and tissue regeneration. In this study, a granular gel (which exhibits ROS scavenging and antibacterial properties) is fabricated based on hyaluronic acid‐*g*‐lipoic acid (HA‐LA). Briefly, HA‐LA is synthesized to fabricate HA‐LA microgels, which are further assembled by Ag^+^ via its coordination effect with disulfide in dithiolane to form a granular gel. The extrudable bulk granular gel possesses a shear‐thinning feature and is immediately restored to a solid state after extrusion, and this can be easily applied to the whole wound area. Therefore, the grafted LA not only allows for the construction of the granular gel but also removes excess ROS from the microenvironment. Additionally, the presence of Ag^+^ realizes the assembly of microgels and has antibacterial effects. In vivo experiments show that the HA‐LA granular gel eliminates excessive ROS at the wound site and up‐regulates the secretion of reparative growth factors, thus, accelerating common and diabetic wound healing significantly. Therefore, the ROS‐scavenging granular gel that can be applied to the wound surface with chronic inflammation demonstrates strong clinical utility.

## 1 INTRODUCTION

Normal wound healing is a complex process that includes the inflammatory, proliferative, and remodeling stages.^1^ These three stages affect each other and overlap during wound healing. It is difficult for chronic wounds (especially those caused by diabetes) to heal in a well‐organized and timely manner compared with normal wounds.^2^ This is because of the imbalance of pro‐inflammatory cytokines, excessive levels of reactive oxygen species (ROS) and proteases on the wound surface, and recurrent bacterial infections at the site of long‐term wounds.^3^, ^4^ Excessive ROS can modify or degrade extracellular matrix (ECM) proteins, promote the increase of protease and inflammatory cytokine levels, inhibit the wound from entering the proliferative stage, prolong the inflammatory response time, and damage the dermal function. All these factors combined can affect the progression of wound healing.^5^, ^6^ Long‐term high blood glucose in diabetic patients is highly conducive to the growth of bacteria, resulting in recurrent bacterial infections on the wound surface and a low level of immune regulation.^4^ The body cannot clear the local infection through the immune system, but there is an excessive inflammatory response because of the influx of immune cells, which damage normal tissues and cells.^7^ Therefore, adequate clearance and regulation of the wound microenvironment is the key to treating chronic refractory diabetic wounds.

Hydrogels used as wound dressings have shown their unique features to significantly promote wound healing. Primarily through materials design, the use of hydrogels as dressings can regulate the immune responses by providing an anti‐inflammatory environment.^8^ Naturally derived macromolecules such as hyaluronic acid (HA, with high molecular weight) could effectively substitute the missing constituents of the ECM and revealed intrinsic anti‐inflammatory characteristics.^9^ Thus, they have been widely used to fabricate immunomodulatory hydrogels to treat chronic wounds.^10^, ^11^ In addition to the natural characteristics of HA that can modulate the features of the immune responses, there is a need to endow the HA molecules with a stronger and more symptomatic immune regulation function towards diabetic wounds.^12^ Lipoic acid (LA), a necessary cofactor of mitochondrial bioenergy enzymes,^13^ contains a dithiolane structure.^14^ This has significant electrophilicity and the ability to react with free radicals, scavenge superoxide and peroxide radicals, and is a potent antioxidant.^15^, ^16^ Owing to long‐term hyperglycemia in diabetic patients, the level of lipid hydroperoxide increases,^17^ leading to the stimulation of the polyol pathway,^18^ the formation of advanced glycation end‐products, and the formation of ROS.^5^, ^19^ LA is approved by the FDA for clinical use to prevent or treat a series of diabetic complications caused by ROS,^20^ eliminate free radicals, and quickly remove ROS in the early stage of inflammation. Additionally, it down‐regulates pro‐inflammatory cytokines to alleviate the inflammatory response.^21^

Injectable hydrogels can be more flexible in adapting to the irregular shapes of wounds and therefore have received more research interest. Significantly, the pre‐crosslinked hydrogels with injectability were attractive during application because they were more convenient and applicable.^22^ The granular gel is a bulk hydrogel formed by densely assembled microparticles.^23^, ^24^ Through the design of particle interaction, the granular gel can exhibit thixotropic properties under shear force, thus allowing for injectability.^25^ Notably, the flowing granular gel can recover to a solid gel immediately after injection.^26^ Thixotropy makes it easy to apply to the whole wound area.^27^, ^28^ Moreover, the inherent porous network structure in assembled microparticles can support cell proliferation, migration, and substance transport.^29^

The present study developed a granular gel system based on HA grafted with LA to regulate the microenvironment of wound inflammation, accelerating wound healing in a safe and multifunctional way. Briefly, HA‐*g*‐LA (HA‐LA) was synthesized and crosslinked by cystamine in a water‐in‐oil emulsion to fabricate HA‐LA microgels. The HA‐LA microgels were assembled using Ag^+^ through its coordination effect with disulfide in dithiolane to form a bulk granular gel. The microstructure, rheological properties, and extrusion performance were then studied. The ability of HA‐LA granular gel to scavenge intracellular ROS and the effect of antibiosis was then evaluated in vitro. The process of wound healing and changes in tissue structure, protein expression, and gene expression was then assessed to evaluate the potential of the HA‐LA granular gel to regulate the wound microenvironment and promote wound healing.

## 2 MATERIALS AND METHODS

### 2.1 Materials

Human umbilical vein endothelial cells (HUVECs) were cultured in ECM cell culture medium. C57BL/6 mice were fed according to the protocol approved by the Laboratory Animal Center of Shanghai Jiao Tong University. HA was purchased from Bloomage Bio. Alpha‐LA, cystamine dihydrochloride (Cys), 4‐(4,6‐dimethoxy‐1,3,5‐triazin‐2‐yl)‐4‐methyl morpholinium chloride (DMTMM), and silver nitrate (AgNO_3_) were purchased from Aladdin. N,N′‐carbonyldiimidazole (CDI), N,N‐dimethylformamide (DMF), formamide (FA), and Span‐80 were purchased from China National Pharmaceutical Group Corporation. ROS Assay Kit was purchased from Beyotime. Dihydroethidium (DHE) were purchased from Sigma‐Aldrich. Streptozotocin (STZ) was purchased from Solarbio. Anti‐mouse F4/80 antibody‐PE, anti‐mouse CD206 antibody‐APC, and anti‐CD31 antibody were purchased from Abcam. Anti‐α‐SMA antibody, anti‐IL‐6 antibody, and anti‐TNF‐α antibody were purchased from Boster Bio. Anti‐VEGF antibody and anti‐integrin α‐3 antibody were purchased from Proteintech. Anti‐IL‐10 antibody was purchased from Affinity. Anti‐IL‐1β antibody was purchased from Santa. All other reagents are commercially available and used directly.

### 2.2 Synthesis and characterization of HA‐LA

#### 2.2.1 Synthesis of HA‐*g*‐LA

First, LA was dissolved in DMF at room temperature, followed by adding CDI. The reaction was stirred at room temperature for 1 hour to fully activate the carboxyl group of LA to obtain lipoyl imidazole (LA‐IM). Then, HA was fully dissolved in FA at 95°C and cooled to room temperature, followed by the addition of catalyst 4‐dimethylaminopyridine and the addition of LA‐IM. After stirring the reaction at room temperature for 4 hours, the crude product was neutralized with an appropriate amount of potassium dihydrogen phosphate solution. After purification by dark dialysis to remove the solvent and impurities produced by the reaction, HA‐LA was collected by freeze‐drying under the condition of avoiding light.

#### 2.2.2 Characterization of HA‐LA

Nuclear magnetic resonance spectroscopy (^1^H NMR): A 5 mg sample was dissolved in deuterated water (D_2_O, 500 μL) with tetramethylsilane (TMS) as the internal standard. The samples were qualitatively and quantitatively analyzed by nuclear magnetic resonance spectrometer (Avance 500 MHz, Bruck, Switzerland) with 128 scans. The degree of substitution was calculated by the software MESTRENOVA after processing the ^1^H NMR results.

### 2.3 Preparation and characterization of HA‐LA microgel (HA‐LA MG)

HA‐LA microgels were prepared by a water‐in‐oil inverse emulsion method. HA‐*g*‐LA was dissolved in deionized water to prepare a solution with a concentration of 5 wt%, and the amidation condensing agent DMTMM and the cross‐linking agent Cys were added. Then it was poured into petroleum ether containing emulsifier Span‐80 and emulsified at 18,000 rev/min. The volume ratio of the mixture, emulsifier, and petroleum ether was 4:1:40. The emulsion was poured into a three‐necked flask containing a large amount of petroleum ether, and the reaction was terminated after 24 hours at room temperature with mechanical stirring at a rotational speed of 500 rev/min. The reaction solution was poured into an equal amount of ethanol absolute, washed 3 to 5 times, and dried in a vacuum to obtain HA‐LA microgels. The morphology of dry and wet microgels was observed by tungsten filament scanning electron microscope (SEM) and phase‐contrast microscope. The particle size and particle size distribution were determined by the software Nano Measurer. Volume swelling ratio was calculated according to the following formula: *S*_v_ = (*V*_1_ − *V*_0_)/*V*_0_ × 100%, where *V*_1_ is the volume of wet microgels and *V*_0_ is the volume of dry microgels.

A certain mass of dry HA‐LA microgels were weighed, the mass recorded as *M*_0_, excess distilled water was added to fully swell the microgels, and centrifuged at 10,000 rev/min. After removing excess water, the weight was measured and recorded as *M*_1_, then the mass swelling ratio (*S*_m_) of the sample was calculated according to the following formula: *S*_m_ = (*M*_1_ − *M*_0_)/*M*_0_ × 100%.

To evaluate the effect of LA grafting ratio and Cys content on mechanical performance of hydrogel, the corresponding bulk hydrogels with different LA grafting ratio and Cys content were prepared (cylindrical samples with a diameter of 8 mm and height of 4 mm) and subjected for compression test on an Instron 5943 testing machine at 10% strain per minute and stopped when hydrogels were broken.

### 2.4 Preparation and characterization of HA‐LA MG‐based granular gel

#### 2.4.1 Preparation of granular gels

A certain mass of dry HA‐LA microgels were weighed and placed in a mold, followed by the addition of silver nitrate solutions with different concentrations to it until the system fully absorbed water to an equilibrium swelling state. After being placed away from light for 30 minutes, HA‐LA microgels were assembled into a bulk gel. The microstructures of dry and wet granular gels were observed by SEM and stereomicroscope.

The total weight *M*_0_ of the dry HA‐LA microgels, the total weight *M*_1_ of the system after adding the silver nitrate solution were recorded, and the water absorption rate Qs (mass ratio) of the sample was calculated according to the following formula: *S*_m_ = (*M*_1_ − *M*_0_)/*M*_0_ × 100%.

#### 2.4.2 Rheological characterization

The rheological properties of the granular gels were tested using a rheometer with a 12 mm flat steel plate fixture. The granular gels were transferred into the rheometer fixture and tested for their modulus‐frequency relationship with a frequency sweep range of 0.1 to 100 Hz and a strain of 0.5%. Strain sweep measurements were performed at a constant frequency of 1 Hz with strain ranging from 0.01 to 1000%. The shear‐thinning behavior was investigated by testing the viscosity change of the granular gels when the shear rate was continuously increased from 0 to 300 s^−1^. The strain step cycled between 1% and 100% at 1 Hz were performed, and the changes of the *G*′ and *G*″ with time and strain were recorded, respectively.

#### 2.4.3 Extrusion performance test

To test the printability and continuity of the granular gels, the prepared granular gels were transferred into a syringe barrel, and an 18 G (inner diameter 0.9 mm) needle was used for extrusion testing. A camera records the extrusion process, and SEM was used to observe the microscopic morphology of the extruded strands. The extruded bars were placed in PBS at 37°C and the system stability after extrusion was recorded.

### 2.5 Evaluation of the subcutaneous degradation of the hydrogel

To evaluate the degradation of the hydrogel, bulk hydrogels (with different Cys content), HA MGs (10 μL), and HA‐LA granular gel (10 μL) were implanted subcutaneously into C57BL/6 mice. The degradation of hydrogels was observed on days 3, 7, and 14 after treatment. Then, the wound tissue was collected, fixed with 4% paraformaldehyde, and sliced with paraffin‐embedded tissue for H&E staining.

### 2.6 Intracellular ROS scavenging experiments

To study the in vitro ROS scavenging ability of the hydrogel, the Reactive Oxygen Species Assay Kit (ROS Assay Kit) was used. First, HUVECs were seeded in a 24‐well plate (200 μL/well, 1 × 10^5^ cells) and cultured for 24 hours until most cells adhered. Next, 5 and 10 mg of HA MGs and HA‐LA granular gel were weighed and mixed with ECM cell culture medium and Rosup (50 mg/mL). This was then added to a 24‐well plate and incubated for 6 hours. The cells were washed (2‐3 times) with a serum‐free cell culture medium before incubation with a ROS probe (DCFH‐DA) for 20 minutes. Fluorescence images were taken using an inverted fluorescence microscope (Nikon, Ti S).

To study the in vivo ROS scavenging ability after treatment with hydrogel, fluorescent probes were used to detect intracellular ROS in wound tissues of normal and diabetic mice collected on day 3 using DHE (Sigma‐Aldrich). Cryosections were incubated with 10 μM DHE at 37°C for 30 minutes and then observed using an inverted fluorescence microscope (Nikon, Ti S) to determine the percentage of the DHE‐stained area. Quantitative data were analyzed by Image J software.

### 2.7 Antibacterial ability test of hydrogel materials

The hydrogel's antibacterial effect was tested using the stiletto implement method. A sterile cotton swab was dipped into the Gram‐positive *Staphylococcus aureus* (*S aureus*) suspension (200 μL; 1 × 10^8^ CFU/mL) and applied evenly on a 9‐mm TSA nutrient Agar medium. Three wells were punched on the TSA nutrient agar medium with a 5‐mm punch. Two of the wells were supplemented with 10 mg of HA MGs and HA‐LA granular gel, and the other was the BLANK group. After a 24‐hour culture, the diameter of the inhibition zone around each well was observed.

### 2.8 Diabetic mice model induced by STZ

After fasting for 12 hours, C57BL/6 mice were induced by intraperitoneal injection of STZ (50 mg/kg) dissolved in sodium citrate buffer (0.1 mol/L, pH 4.5) once a day for a total of five injections.^30^ One week after the injection, the mice with fasting blood glucose levels >16.7 mmol/L for 2 successive days were defined as diabetic mice.

### 2.9 Accelerating wound closure by applying hydrogel materials

C57BL/6 mice were purchased from Shanghai Jihui Experimental Animal Breeding Co., Ltd. Eight‐week‐old SPF‐grade male mice weighing around 20 to 25 g were randomized into different experimental groups. A total of six independent biological replicates were performed for each group to confirm the findings.

The in vivo wound healing induced by the hydrogel was performed on wound models in healthy C57BL/6 mice (NM) and STZ‐induced diabetic C57BL/6 mice (DM). First, the back hair of normal and diabetic C57BL/6 mice was removed, and two 5‐mm diameter full‐thickness skin defects were made on each side of the midline on the back using a 5‐mm punch. A round silicone sheet was sewn around each wound, and the HA MGs (CON.) and HA‐LA granular gel (EXP.) were applied (dressings changed every 3 days).^31^ The wounds were randomly divided into six groups: NM (BLANK), NM (CON.), NM (EXP.), DM (BLANK), DM (CON.), and DM (EXP.) groups. Observations, measurements, and analyses were carried out on days 3, 7, and 14 using ImageJ software. The simulated diagram of wound healing was drawn with Adobe Illustrator (2020), depicting photos of wounds taken on different days during the general observation.

### 2.10 Evaluation of changes in the wound microenvironment

Changes in the wound microenvironment were observed on days 3, 7, and 14 after treatment. First, the wound tissue was collected, fixed with 4% paraformaldehyde, and sliced with paraffin‐embedded tissue for H&E and Masson's trichrome staining (MTS). Anti‐CD31 antibody (Abcam, ab182981, 1:2000), anti‐α‐SMA antibody (Boster Bio, bm0002, 1:1000), anti‐integrin α‐3 antibody (Proteintech, 66,070‐1‐ig, 1:500) were incubated on tissue sections. This was followed by staining with the corresponding fluorescence‐labeled secondary antibodies and the cell nuclear dye (DAPI). The results were observed using an inverted fluorescence microscope (Nikon, Ti S) and quantitatively analyzed using ImageJ software.

### 2.11 RNA‐sequencing analysis

Total RNA was isolated from the wound tissues of normal and diabetic mice and collected on day 7. RNA sequencing samples were obtained using TRIzol reagent according to the manufacturer's instructions. Briefly, wound tissues (100 mg) were harvested, treated with TRIzol reagent (1 mL), and homogenized with a tissue homogenizer. After adding chloroform (200 μL), the mixture was shaken and mixed evenly. After centrifugation, the supernatant was removed, followed by the addition of isopropanol to precipitate RNA. Then, 75% ethanol solution was added for washing, and the precipitate was retained after centrifugation. Diethyl pyrocarbonate water was added to dissolve the RNA. The RNA‐seq Library Preparation Kit (Illumina) was used to construct the cDNA library. The cDNA terminal was repaired, the connector was joined, the product was purified, and the fragment size was sorted. After the cDNA library was generated, it was sequenced on an Illumina HiSeq 4000 platform. FastQC was used for quality evaluation, HISAT2 was used for sequencing evaluation, and String Tie was used for gene structure analysis. Ballgown software was used to quantify transcription levels and identify differentially expressed genes, and *P* < .05 was adjusted as a cutoff value. The Gene Ontology functional and Kyoto Encyclopedia of Genes and Genomes pathway enrichment analysis were performed using the Database for Annotation for differentially expressed genes.

### 2.12 Statistical analysis

The statistical analysis of the data was evaluated by GraphPad Prism 9 software (GraphPad Software Inc., San Diego, CA, USA). The data were expressed as mean ± standard deviation (SD) and compared by *t*‐test. All the experiments were repeated three times. *P* < .05 were considered statistically significant.

## 3 RESULTS AND DISCUSSION

### 3.1 Preparation and characterization of HA‐LA microgels (HA‐LA MGs)

**FIGURE 1:**
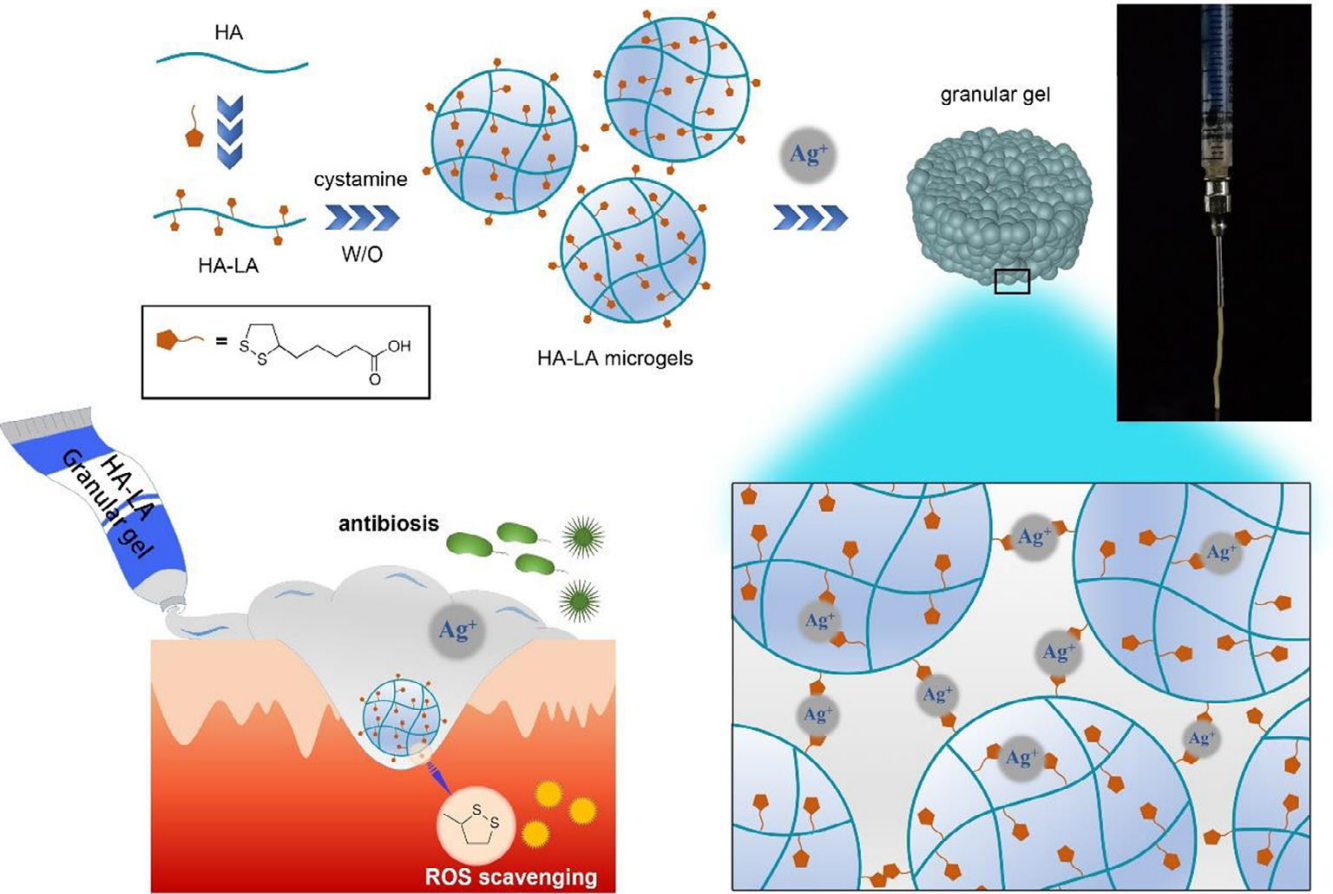
Schematic diagram of HA‐LA granular gel preparation and functions. HA‐LA, hyaluronic acid‐*g*‐lipoic acid

**FIGURE 2:**
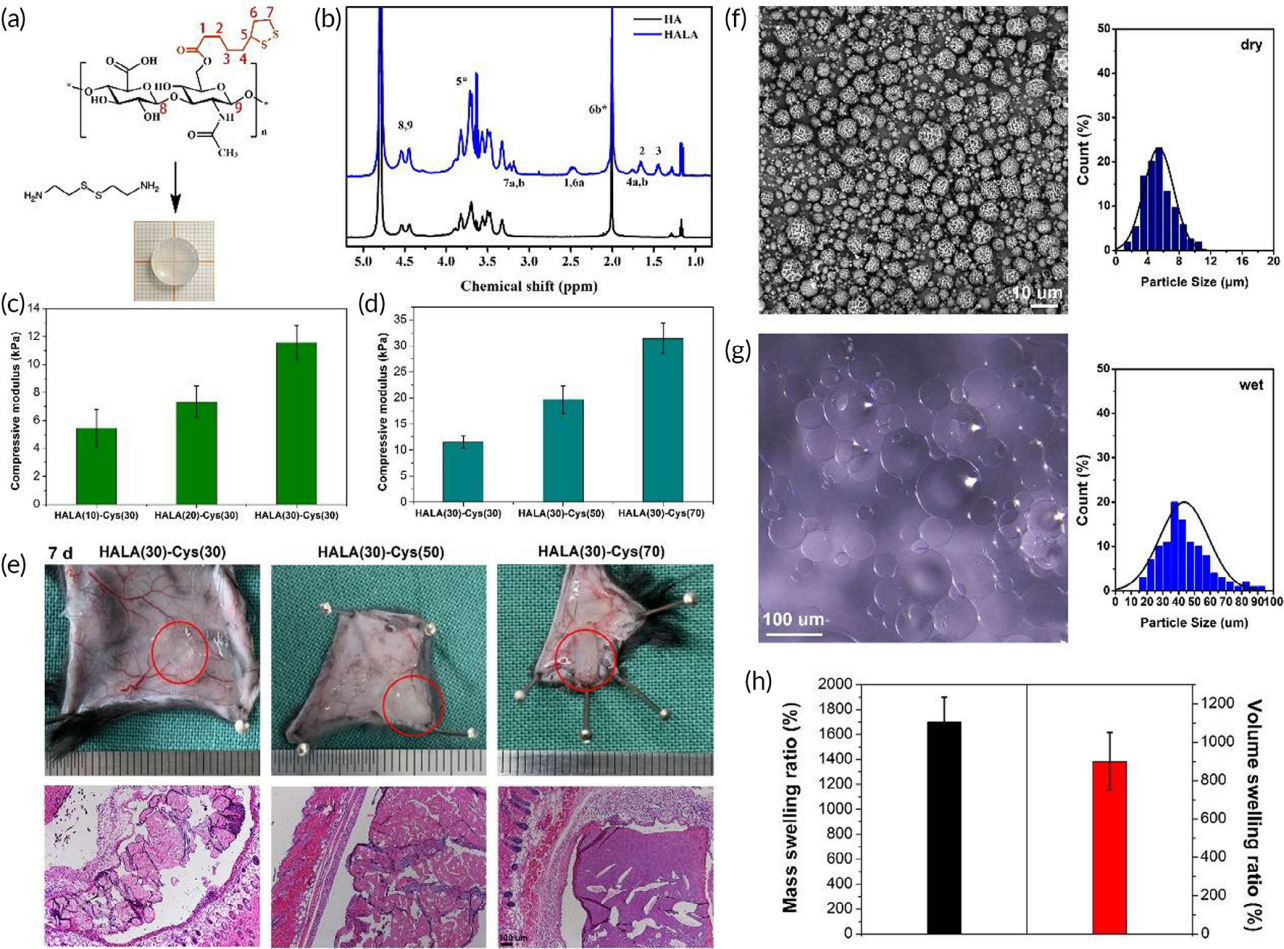
Preparation and characterization of HA‐LA bulk hydrogel and microgels. (a) Modified hyaluronic acid: HA‐LA. (b) ^1^H NMR proved that HA‐LA was successfully synthesized. (c) Compressive moduli of hydrogels with different LA grafting rate (HA‐LA (lipoic acid grafting rate)‐Cys (cross‐linking density)). (d) Compressive moduli of hydrogels with different crosslinking density (HA‐LA(lipoic acid grafting rate)‐Cys(cross‐linking density)). (e) General observation and H&E staining of hydrogels degradation on 7 day post‐implantation (scale bar = 100 μm, n = 3). (f) The SEM image of HA‐LA microgels and the diameter statistics (scale bar = 10 μm). (g) The swelling microspheres after water absorption observed by phase‐contrast microscope and the diameter statistics (scale bar = 100 μm). (h) Water absorption and volume expansion of microgels. Cys, cystamine dihydrochloride; HA‐LA, hyaluronic acid‐*g*‐lipoic acid; NMR, nuclear magnetic resonance; SEM, scanning electron microscopy

HA‐LA, a hyaluronic acid macromolecule grafted with LA, was synthesized (Figure 1). ^1^H NMR confirmed that the HA macromolecule was successfully grafted with LA (Figure 2a,b). According to Figure S1 and Table S1, the *M*_w_ of HA was 4.4 × 10^4^ g/mol, while after LA grafting, the *M*_w_ of HA‐LA reduced significantly to 2.3 × 10^4^ g/mol.

The LA grafting rate and crosslinking degree were optimized to fabricate the bulk hydrogel for the compression test. Here, synthesized HA‐LA was crosslinked by cystamine via the amidation reaction between the terminal amino group of cystamine and the carboxyl group of HA‐LA (Figure 2c). The compressive modulus was found to increase with the LA modification rate, which might be related to the hydrophobic feature of 1,2‐dithiolane. However, HA‐LA with a grafting rate of LA higher than 30% was easier to precipitate during dialysis and was more challenging to dissolve in the water again. Because LA was involved in ROS scavenging to maintain water solubility with a high level of LA, the present study controlled the grafting rate of LA to 30%.

The effect of crosslinking density (cystamine content) on hydrogel mechanical performance was also evaluated. With the increase of cystamine content, the crosslinking density increased, leading to a significant increase of compressive moduli (Figure 2d). However, because of the high swelling behavior of HA‐based polymers, a crosslinking density lower than 30% would lead to insufficient crosslinking, while higher crosslinking density (over 50%) significantly decreased the degradation rate of the hydrogel. Figure 2e shows that 7 days post‐implantation, a hydrogel with a crosslinking density of 30% was significantly swollen. H&E staining showed hydrogel degradation with a loose internal structure. However, a hydrogel with a crosslinking density higher than 50% showed slight swelling and a significantly more compact structure. Typically, the disulfide bond can be readily cleaved because glutathione is widely present in tissues.^32^ However, the cystamine's high content and dithiolane presence might cause an extensive disulfide exchange reaction, leading to a prolonged degradation. Thus, to minimize the residue of materials in the body, a hydrogel with a crosslinking density of 30% was used in the subsequent studies.

Based on the above optimization, HA‐LA microgels were fabricated in a water‐in‐oil inverse emulsion, which was also crosslinked by cystamine. Microgels with relatively regular spherical shapes were obtained (Figure 2f,g). The diameter distribution of the dry microspheres was 1 to 11 μm. After absorbing water, the microspheres underwent noticeable volume swelling, and the diameter range was 15 to 100 μm. The mass swelling ratio of HA‐LA microgels was about 1700%, while the average volume swelling ratio was about 900% (Figure 2h).

### 3.2 Preparation and characterization of HA‐LA granular gel

**FIGURE 3:**
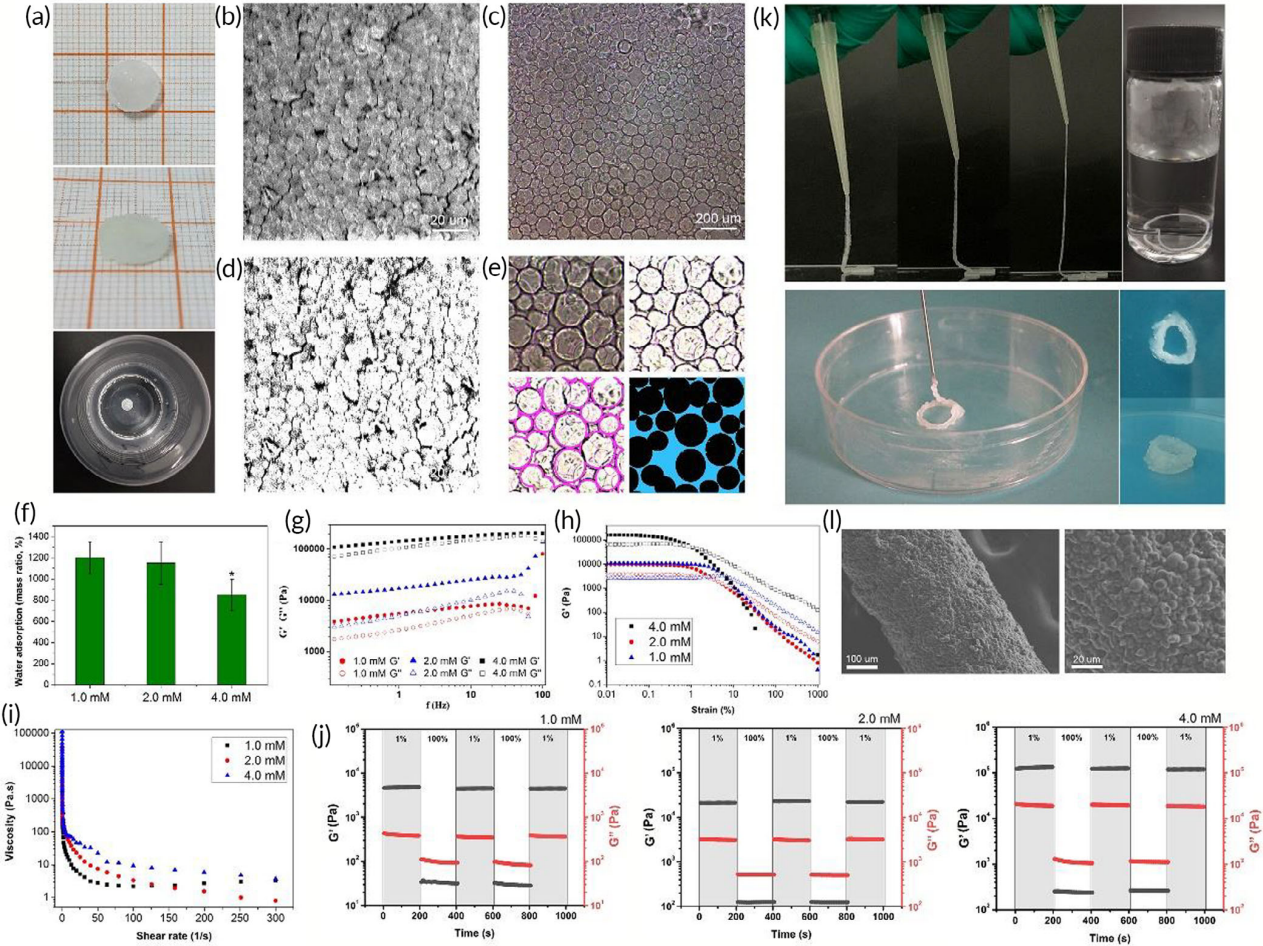
Preparation and characterization of granular gel. (a) HA‐LA microgels were assembled by silver ions through coordination to form bulk granular gels, which were stable in water. (b) The SEM image of dry granular gel (scale bar: 20 μm). (c) The wet granular gel observed by stereomicroscope (scale bar: 200 μm). (d) SEM image processing to show gap among microgels at dry state. (e) Image processing to show spaces among microgels at wet state (pink lines were used to draw the outline of the microspheres; the blue area indicated the gap among microgels). (f) Water absorption of granular gel prepared with different concentrations of Ag^+^. (g) The rheological test confirms that it is a solid‐state hydrogel. (h) The rheological test showed yield when the shear force reaches a certain degree. (i) The rheological test showed shear‐thinning feature. (j) Evaluation of self‐recovery of the granular gels under alternating strains of 1% and 100%. (k) The extrusion test and the stability of extruded filament in PBS, and the general observation of extruded granular gel with solid state. (l) The SEM images of extruded filament with densely packed microgels (scale bar: 100 μm for left, 20 μm for right). The data represent the mean ± SD. * indicates that these data showed significant differences with other data. HA‐LA, hyaluronic acid‐*g*‐lipoic acid; SEM, scanning electron microscopy

By simply treating the dry HA‐LA microgel particles with Ag^+^ solutions, the bulk granular gels formed by coordinating Ag^+^ and disulfide in dithiolane could be obtained.^33^ These HA‐LA microgel particles showed good stability in PBS (Figure 3a). The microstructure of the granular gel in dry and wet states was observed through SEM and the stereoscopy microscope (Figure 3b,c). It was found that the microgels inside the granular gel were densely packed, but there were still a large number of gaps, which provided a guarantee for matter exchange and cell migration (Figure 3d,e). Image analysis (using ImageJ) showed that the area occupied by gaps was 13.2% ± 1.1%, which was not affected by the concentration of Ag^+^. This was one of the characteristics of granular gel that was different from traditional injectable hydrogels. The Ag^+^ concentration was changed to prepare three groups of granular gel. By evaluating their water absorption rate, it was found that a higher concentration of Ag^+^ led to a lower water absorption rate, revealing strong and stable forces between microgels (Figure 3f). However, the higher concentrations of Ag^+^ might have also resulted in higher tissue toxicity. Therefore, according to the literature, an Ag^+^ concentration of 2 mM was selected in this study.^34^

Using a rheological evaluation, it was found that the storage moduli of the granular gel samples were greater than their loss moduli when the frequency sweep range was 0.01 to 100 Hz, confirming that the granular gel was an elastic bulk gel. Additionally, the granular gel with a higher Ag^+^ concentration showed a greater storage modulus (Figure 3g). The results confirmed that the HA‐LA microgels could be assembled by coordinating Ag^+^ to construct the elastic solid bulk granular gel.

Notably, as long as there was an increase in strain, *G*′ began to decrease, while *G*″ increased rapidly. When *G*′ was equal to *G*″, the breaking points of the granular gel were achieved (Figure 3h). When the strain kept exceeding the breaking point, both *G*′ and *G*″ decreased, but *G*″ was greater than *G*′. Moreover, the granular gel showed a shear‐thinning feature.^35^ According to Figure 3i, the viscosity of the granular gel significantly decreased with the increase in shear rate. Under shear force, the bulk granular gel structure was destroyed. With the friction and slippage among the internal microgels, the granular gel switched from the original “solid” state with high viscosity to the “fluid” state with low viscosity, similar to the liquid. In addition, Figure 3j shows that the three groups of granular gels maintained their moduli after cyclic low‐high strain action. Under low strain, *G*′ was more significant than *G*″. When the strain increased to 100%, the hydrogel was broken, *G*′ decreased quickly and was lower than *G*″. When returning to the initial 1% strain, *G*′ immediately recovered without significant decrease, indicating the self‐healing property of the granular gels.

The above rheological test results confirmed that the granular gel was extrudable. Extrusion experiments further confirmed this property. According to Figure 3k, the granular gel could be easily extruded by injection, and the extruded filament had excellent continuity. The extruded strips immersed in PBS were found to maintain a stable shape, indicating that the granular gels maintained the elastic characteristics of the solid gel after extrusion. In addition, it was worth noting that the extruded gel immediately recovered to a solid state after the removal of shear force, performing well in plasticity and shape stability. The microstructure of the extruded filament was further observed using SEM, showing that the extruded filament was assembled and stacked by many microspheres (Figure 3l). In addition, the rheological tests and extrusion experiments illustrated another feature of the granular gel. The gel behaved as a stable solid when there was no shear force. Under the action of shear force, the microgels in the system slide relative to each other, showing flow dynamics. After removing the shear force, the gel returned to a solid state. Therefore, the present granular gel could be easily extruded and applied to wounds, making the clinical application operation more convenient.

### 3.3 Evaluation of the ROS‐scavenging, antibacterial ability, and degradation tests for the HA‐LA granular gel

**FIGURE 4:**
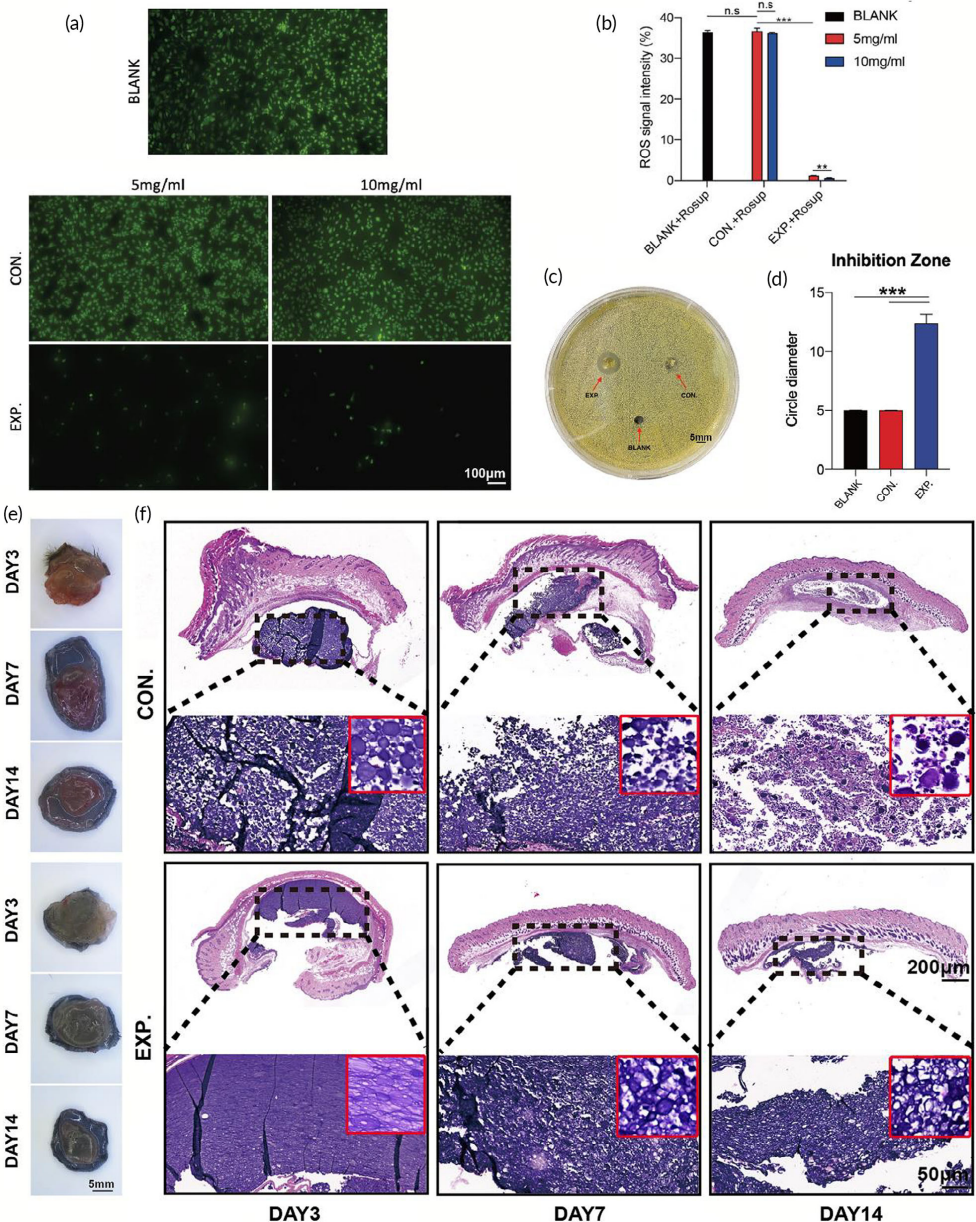
Evaluation of ROS‐scavenging, antibacterial effect, and degradation of granular gel. (a) Fluorescence images and the statistical data (b) of different concentrations of HA MGs and HA‐LA granular gel co‐cultured with HUVECs cells, with the addition of reactive oxygen stimulant (Rosup) (scale bar: 100 μm, n = 3). (c) Antibacterial test of the three groups co‐cultured with *Staphylococcus aureus* (*S aureus*) (scale bar: 5 mm, n = 3). (d) Quantification of bacterial inhibition diameter in the three groups co‐cultured with *S aureus*; General observation (e) and H&E staining (f) of HA MGs and HA‐LA granular gel on days 3, 7, and 14 (CON. = treated with HA MGs; EXP. = treated with HA‐LA granular gel) (scale bar = 200 μm, 50 μm, n = 3). The data represent the mean ± SD. ***P < .001, according to *t*‐test. HA‐LA, hyaluronic acid‐*g*‐lipoic acid; HA MGs, hyaluronic acid microgels; HUVECs, Human umbilical vein endothelial cells; SD, standard deviation

The ability of the HA‐LA granular gel to remove intracellular ROS was evaluated using the strong impact of ROS on tissue repair. HUVECs were cultured in 24‐well plates (200 μL/well, 1 × 10^5^ cells) with an endothelial cell medium containing 1 μl Rosup (50 mg/ mL) that can induce ROS production in cells. HA microgels (HA MGs, 5 mg/mL, 10 mg/mL, and CON. group) and HA‐LA granular gel (5 mg/mL, 10 mg/mL, and EXP. group) were added to each well for incubation for 6 hours. Then the cells were stained with DCFH‐DA cell dye with a ROS‐specific probe. According to the representative images and quantitative detection of fluorescence intensity in Figure 4a,b, cells in the CON. group showed strong fluorescence intensity. The two concentrations of HA MGs (5 mg/mL and 10 mg/mL) showed no significant difference in fluorescence intensity. However, cells in the EXP. group showed significantly lower fluorescence intensity than those in the CON. group. In addition, compared with the 5 mg/mL HA‐LA granular gel treatment, the fluorescence intensity of cells in the EXP. group with 10 mg/mL HA‐LA granular gel treatment decreased significantly. There was also a BLANK group with no treatment, showing similar fluorescence intensity of cells with the CON. group. The comparison between the BLANK and CON. groups indicated that HA MGs showed no significant ROS‐scavenging ability in vitro. In contrast, the HA‐LA granular gel exhibited a strong ROS‐scavenging ability, which increased with the granular gel concentration.

Ag^+^ was not only used to assemble HA‐LA MGs but also possessed a specific antibacterial effect.^36^ The antibacterial ability of HA‐LA granular gel was evaluated using the antibacterial test. According to Figure 4c,d, it was found that the BLANK group (with no treatment) and the CON. group (treated with HA MGs) had almost no inhibition zone around the hole. The bacterium, *S aureus*, grew evenly around the hole, and the circle diameter was the same as the diameter of the initial hole punch (5 mm). In the EXP. group (treated with HA‐LA granular gel), an apparent inhibition zone was formed around the hole, with an average diameter of 12.5 mm. The results show that HA‐LA granular gel exhibited an apparent inhibitory effect on the growth of *S aureus*.

To better understand the degradation of hydrogels, 10 μL of HA MGs and HA‐LA granular gel were injected subcutaneously into C57 mice, respectively, and observed on days 3, 7, and 14. Figure 4e shows that the degradation extent of the two hydrogels was similar from day 3 to day 14, and there was no apparent absorption on day 3. On day 7, the volume of the two hydrogels significantly shrunk, and on day 14, the volume reduction was more pronounced, but residual hydrogels were still not entirely absorbed. In H&E staining, Figure 4f shows that LA was further crosslinked because of the mutual coordination of silver ions, resulting in denser compacted microspheres in the EXP. group (HA‐LA granular gel). The CON. group (HA MGs) also had more internal pores than the EXP. group on day 3. On day 7, the two hydrogels were degraded to a certain extent, and the internal structure was looser compared with day 3. On day 14, the internal structure of the HA‐LA granular gel in the EXP. group was looser compared with that on day 7. In the CON. group, the internal structure was looser than that in the EXP. group, exhibiting many lymphocytes, erythrocytes, and macrophages around the HA MGs. Most HA MGs were absorbed organically, but some were not degraded completely and remained, which was consistent with the general observation. Overall, although there was little difference between the two hydrogels in the general observation; it was apparent that the degradation rate of HA MGs was faster than that of the HA‐LA granular gel in H&E staining.

### 3.4 Normal and diabetic wound healing with dressing treatment

Based on the findings that the HA‐LA granular gel possessed ROS‐scavenging and antibacterial properties, the HA‐LA granular gel was then applied to normal and diabetic C57 mice wounds (EXP. Group). The application of HA microgels (HA MGs) to the wound was defined as the CON. group. Therefore, the experimental subjects could be divided into six groups: NM (BLANK), NM (CON.), NM (EXP.), DM (BLANK), DM (CON.), and DM (EXP.) group. Then the wound‐healing effect was monitored on normal C57 mice (NM) and diabetic C57 mice (DM) in vivo.

**FIGURE 5:**
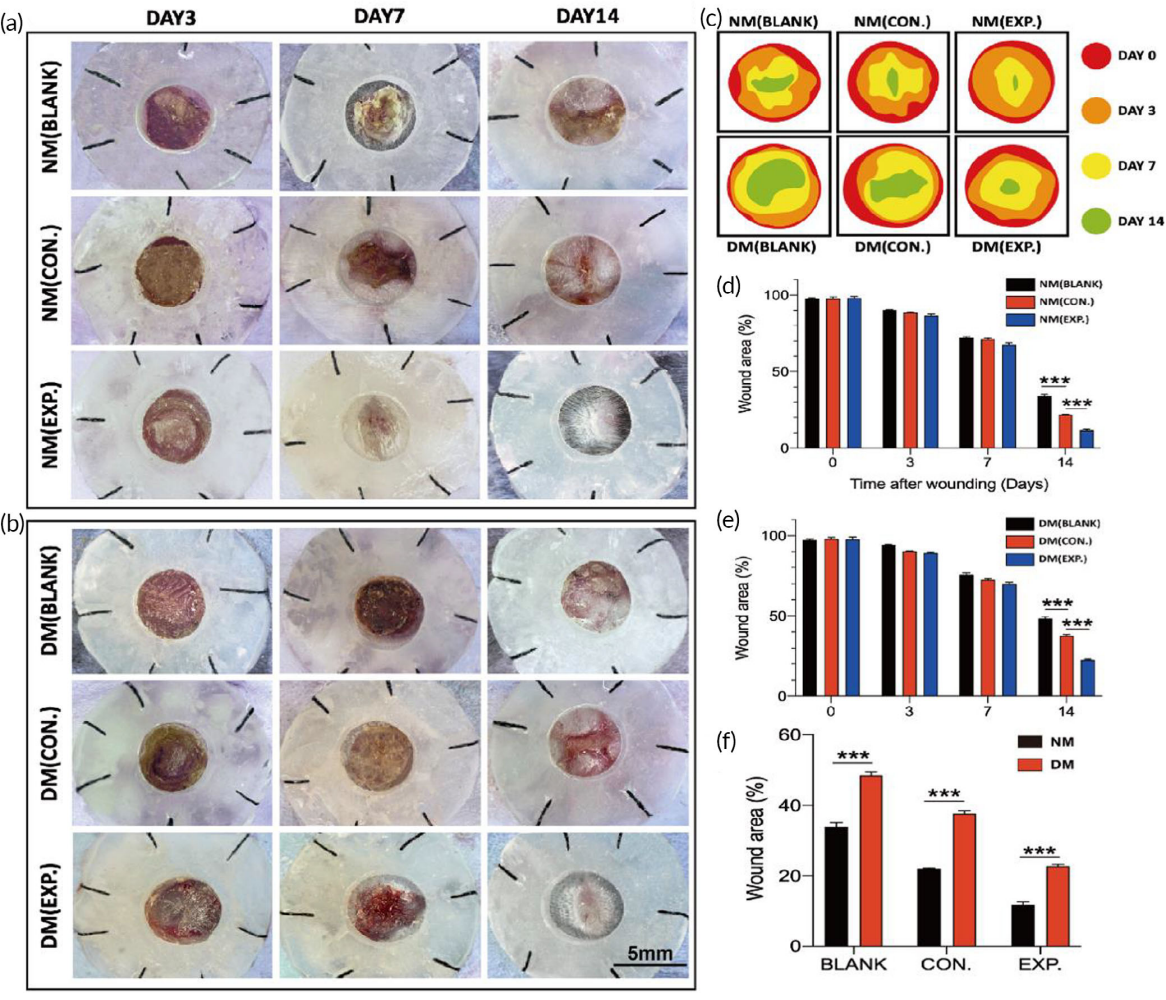
General observation of different dressings on the wound of normal and diabetic C57 mice. (a, b) General observation images of wound treatment with different dressings on days 3, 7, and 14 (NM (CON.) = normal mice wound treated with HA MGs, NM (EXP.) = normal mice wound treated with HA‐LA granular gel, DM (CON.) = diabetic mice wound treated with HA MGs, DM (EXP.) = diabetic mice wound treated with HA‐LA granular gel) (scale bar: 5 mm, n = 3). (c) Simulated diagram of wound healing. (d) Quantification of wound residual area rate on days 0, 3, 7, and 14 in normal C57 mice. (e) Quantification of wound residual area rate on days 0, 3, 7, and 14 in diabetic C57 mice. (f) Quantification of wound residual area rate on day 14 in normal and diabetic C57 mice. The data represent the mean ± SD. ***P < .001, according to *t*‐test. HA‐LA, hyaluronic acid‐*g*‐lipoic acid; HA MGs, hyaluronic acid microgels; SD, standard deviation

Figure 5a‐f shows that the healing of the three groups of normal mice was similar on day 3. The scab and the wound contraction appeared at the wound site. The relative area of the wound was 85% to 90% of the initial wound area. While the three groups of diabetic mice had slightly worse healing than normal mice, the relative area of the wound was about 90% of the initial wound area. On day 7, the scab had covered the wound of the normal mice, and there was no significant healing difference in the three groups of normal mice. The relative area of the wound was about 70% of the initial wound area. The healing trend of the three groups of diabetic mice was similar to the normal mice, showing the relative area of the wound to be about 75% of the initial wound area. On day 14, significant differences were observed in the normal mouse group, and the NM (EXP.) group healed better than the other two groups, which were completely healed. Additionally, the color and texture of the wound were similar to normal skin, and the relative wound area of the NM (BLANK) and the NM (CON.) was 35% and 20% of the initial wound area, respectively. Diabetic mice also showed better healing results in the DM (EXP.) compared with the other two groups. The relative wound area in the DM (EXP.) was about 25% of the initial wound area, while the relative wound area of the DM (BLANK) and the DM (CON.) was 50% and 40% of the initial wound area, respectively (Figure 5a‐f). In short, applying the HA‐LA granular gel on the wound surface significantly promoted both normal and diabetic mouse wound healing.

**FIGURE 6:**
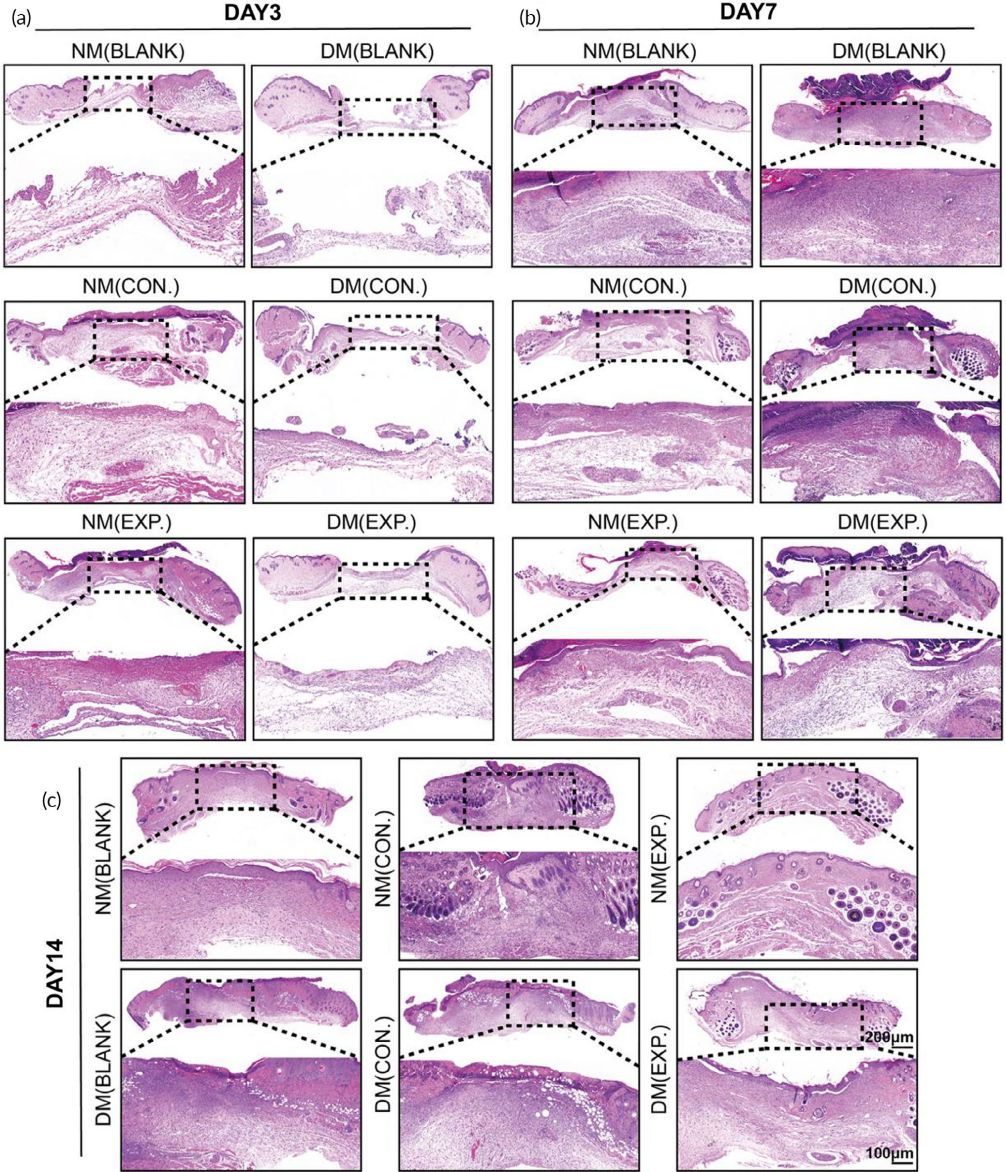
Histology evaluation (H&E staining) of wound healing. (a) H&E staining of the NM (BLANK), NM (CON.), NM (EXP.), DM (BLANK), DM (CON.), and DM (EXP.) groups on day 3, (b) day 7, and (c) day14 (NM (CON.) = normal mice wound treated with HA MGs, NM (EXP.) = normal mice wound treated with HA‐LA granular gel, DM (CON.) = diabetic mice wound treated with HA MGs, DM (EXP.) = diabetic mice wound treated with HA‐LA granular gel) (scale bar: 100 μm and 200 μm, n = 3). HA‐LA, hyaluronic acid‐*g*‐lipoic acid; HA MGs, hyaluronic acid microgels

To further investigate the effect of different dressings on wound healing, H&E staining and MTS were carried out to evaluate the histological differences in the wound tissue. On day 3, there were apparent skin lesions in all groups. In the discontinuity of the sarcolemma under the squamous epithelium, many lymphocytes and neutrophils infiltrated the tissue, and the fat was liquefied, showing a vacuolar appearance. There were more new granulation tissues on the wounds of the NM (CON.) group and the NM (EXP.) group, indicating that the presence of HA MGs and HA‐LA granular gel positively affected normal mice wounds. However, almost no new granulation tissues were observed in the DM (BLANK) and DM (CON.) groups, while more new granulation tissue on the diabetic mice wounds treated with HA‐LA granular gel was observed (Figure 6a).

On day 7, there were no significant differences in the formation of granulation tissue among the groups of normal mice. In groups of diabetic mice, granulation tissue formation was observed in the DM (BLANK) and DM (CON.) groups and had more neutrophils and lymphocytes, revealing that the wounds were still at the inflammatory stage. The healing progression was delayed when compared with normal mice wounds. However, the DM (EXP.) group had more new granulation tissue, and the inflammatory reaction was milder than that in the DM (BLANK) and DM (CON.) groups. In addition, the NM (EXP.) group had already formed a completely new epidermis at the wound site, which was thicker and more complete than those in the NM (BLANK), NM (CON.), and DM (EXP.) groups. In contrast, almost no new epidermis was observed in the DM (BLANK) and DM (CON.) groups (Figure 6b). On day 14, although the thickness of the new epidermis was varied, the new epidermis was observed in all groups. Compared with the DM (BLANK) and DM (CON.) groups, the new epidermis was thicker and more complete in the DM (EXP.) group of diabetic mice. New appendages such as sweat glands and hair follicles were observed in the NM (EXP.) group. In contrast, the new epidermis of the DM (BLANK) and DM (CON.) groups were relatively thin and incomplete, and the scabs were not completely exfoliated (Figure 6c). Besides, few residual microgels could be found embedding in regenerated tissues (Figure S2).

**FIGURE 7:**
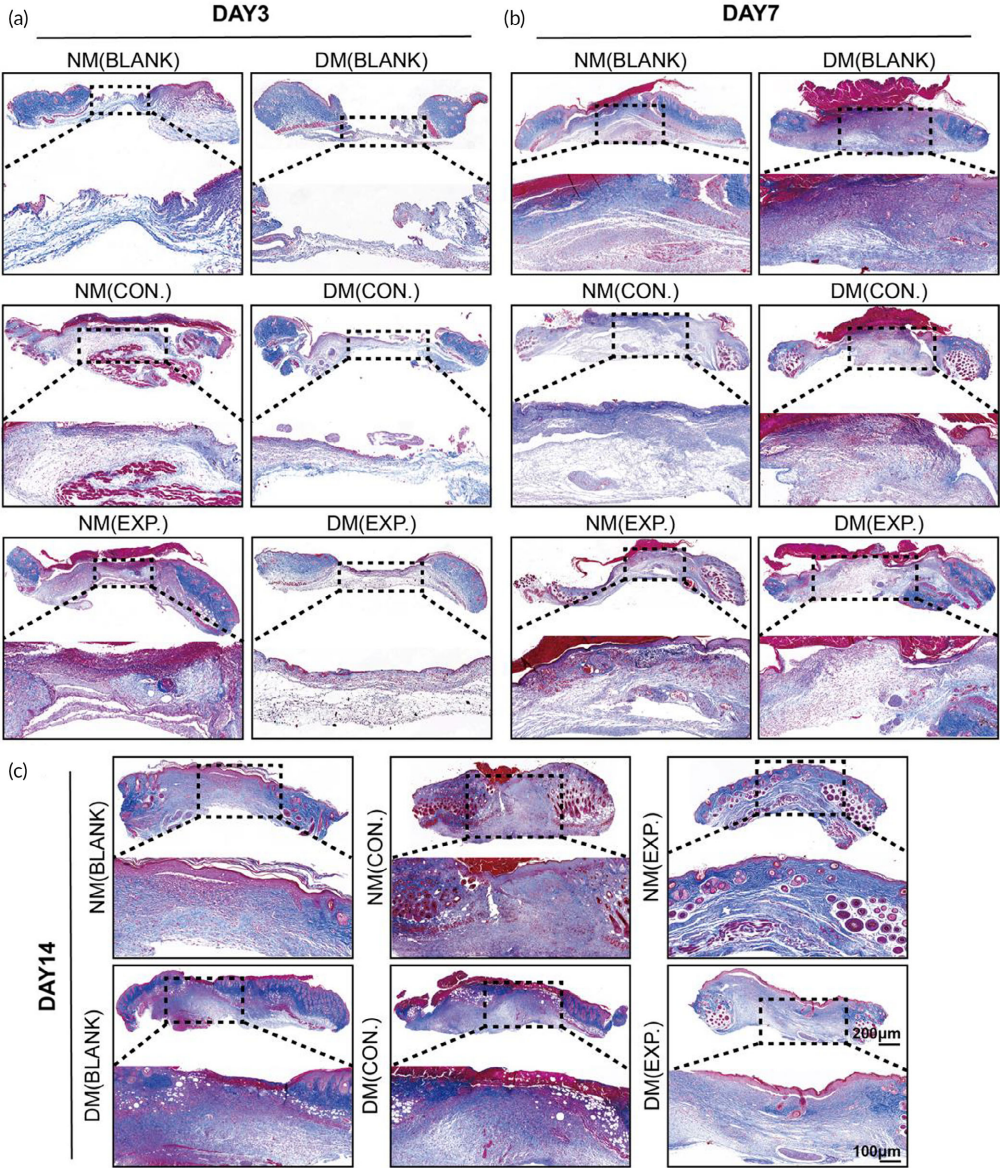
Histology evaluation (Masson staining) of wound healing. (a) Masson staining of the NM (BLANK), NM (CON.), NM (EXP.), DM (BLANK), DM (CON.), and DM (EXP.) group on day 3, (b) day 7, and (c) day 14 (NM (CON.) = normal mice wound treated with HA MGs, NM (EXP.) = normal mice wound treated with HA‐LA granular gel, DM (CON.) = diabetic mice wound treated with HA MGs, DM (EXP.) = diabetic mice wound treated with HA‐LA granular gel) (scale bar: 100 μm and 200 μm, n = 3). HA‐LA, hyaluronic acid‐*g*‐lipoic acid; HA MGs, hyaluronic acid microgels

According to Masson staining, on day 3, collagen deposition in the wounds was not dense, and there was no apparent difference in each group. The fibrous collagen structure could not be observed (Figure 7a). On day 7, the three groups of normal mice showed denser collagen deposition when compared with those on day 3. The DM (EXP.) group of diabetic mice also had denser collagen deposition than the DM (BLANK) and DM (CON.) groups. The fibrous collagen structure could be observed at the same time. Collagen fibers at the wound surface of the NM (EXP.) group dispersed more evenly with higher density than that of the NM (BLANK), NM (CON.), and DM (EXP.) groups. However, observing collagen fibers in the DM (BLANK) and DM (CON.) groups was challenging. Combined with the results of H&E staining, it can be seen that the DM (BLANK) and DM (CON.) groups were still in the inflammatory stage. The inflammatory change of ECM might interfere with the staining of collagens resulting in the relatively red and dark blue change in Masson staining (Figure 7b). On day 14, the collagen deposition in all groups increased compared with day 7. Collagen fibrous structures were observed in all wounds, and it was worth noting that the NM (EXP.) group had a higher degree of ordered structure (Figure 7c).

In summary, the healing of the normal mice in the three groups was better than that of the diabetic mice. The NM (EXP.) group showed the closest tissue to normal skin tissue on day 14, while the healing of the DM (EXP.) group was much better than that of the DM (BLANK) and DM (CON.) groups, which was approximately the same as that of the NM (CON.) group. The results indicated that applying the HA‐LA granular gel to the wound could significantly stimulate the repair of wound tissue and promote wound healing. Overall, the histological results were consistent with the general observation.

### 3.5 Assessment of in vivo ROS‐scavenging and tissue regeneration after applying HA‐LA granular gel to wounds

**FIGURE 8:**
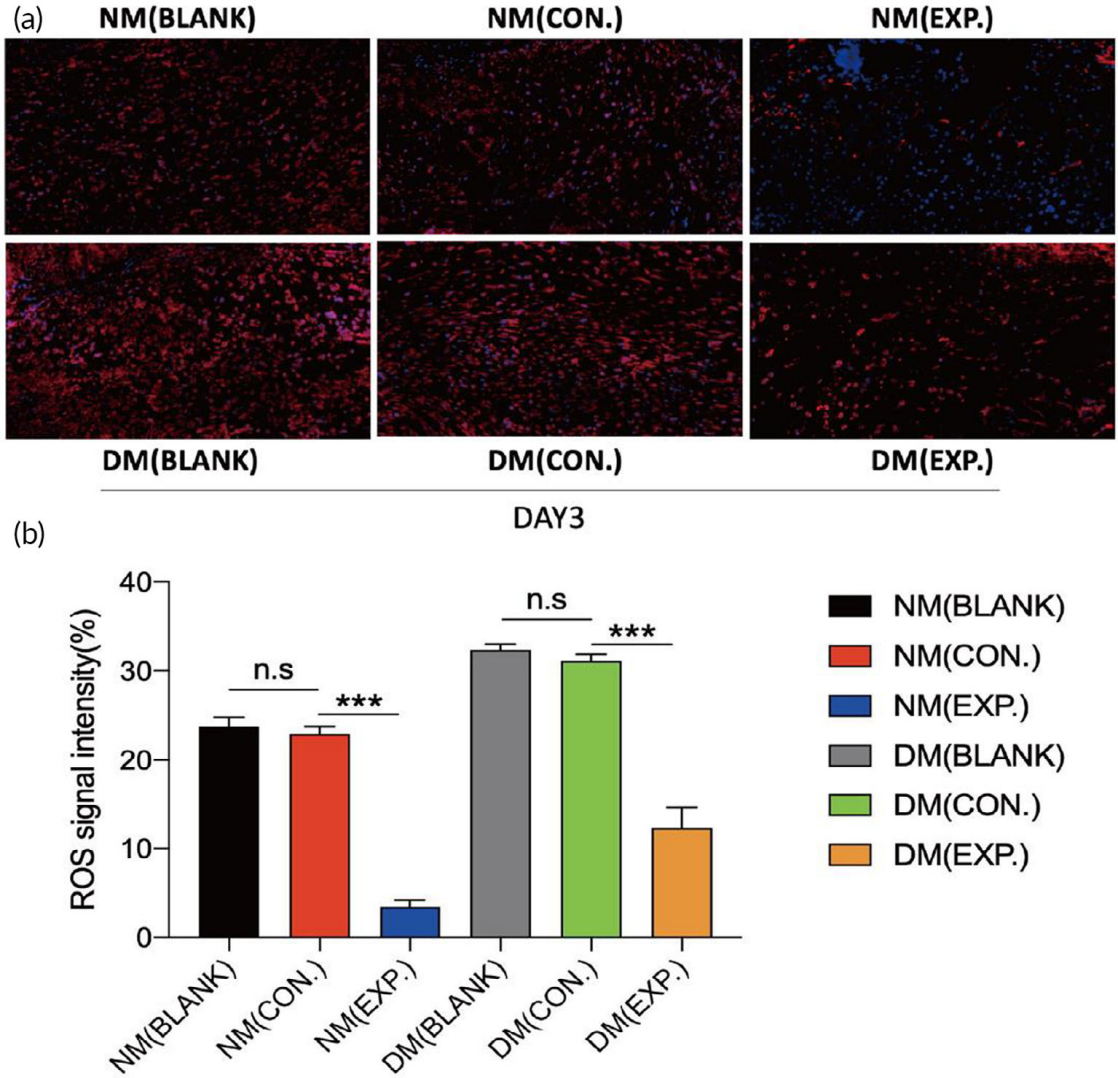
In vivo evaluation of ROS‐scavenging ability of HA MGs and HA‐LA granular gel. (a) Fluorescence images and the statistical data (b) of dihydroethidium (DHE) from different groups on day 3; DAPI (blue) staining of nuclei (NM (CON.) = normal mice wound treated with HA MGs, NM (EXP.) = normal mice wound treated with HA‐LA granular gel, DM (CON.) = diabetic mice wound treated with HA MGs, DM (EXP.) = diabetic mice wound treated with HA‐LA granular gel) (scale bar: 50 μm, n = 3). The data represent the mean ± SD. ***P < .001, according to *t*‐test. HA‐LA, hyaluronic acid‐*g*‐lipoic acid; HA MGs, hyaluronic acid microgels; SD, standard deviation

The in vivo removal of ROS was further assessed. Immunofluorescence staining of DHE was performed on the skin wound tissue on day 3. Figure 8a,b shows that the fluorescence density of the NM (EXP.) group was the lowest among all groups. There was no statistical difference between the fluorescence intensity of the NM (BLANK) and NM (CON.) groups. Similarly, there was no statistical difference in the fluorescence intensity between the DM (BLANK) and DM (CON.) groups. However, the skin tissue of diabetic mice treated with HA‐LA granular gel showed a lower level of ROS, and there was a significant difference between the two groups and the DM (EXP.) group. The results indicated that HA MGs showed no significant ROS‐scavenging ability in vivo, while the HA‐LA granular gel exhibited a robust ROS‐scavenging ability. Overall, the in vivo DHE immunofluorescence staining results were consistent with the in vitro DCFH‐DA immunofluorescence staining results.

**FIGURE 9:**
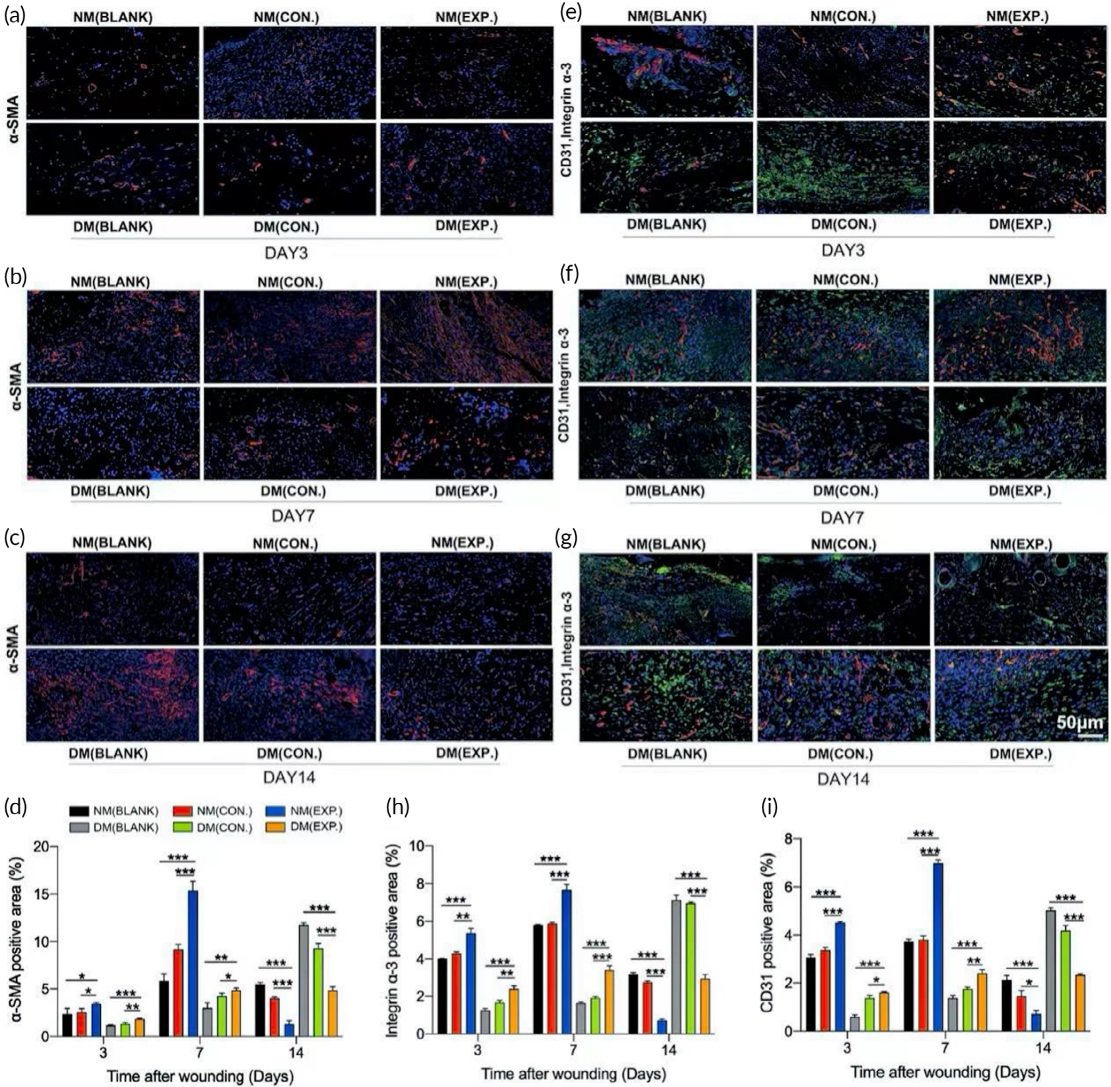
Wound microenvironment changes (healing) induced by ROS‐scavenging granular gel. (A‐D) Immunofluorescence images (A‐C) and the statistical data (D) of α‐SMA (red) from different groups on days 3, 7, and 14. (E‐I) Immunofluorescence images (E‐G) and the statistical data (H and I) of CD31 (red) and integrin α‐3 (green) double‐stained sections from different groups on days 3, 7, and 14; DAPI (blue) staining of nuclei (NM (CON.) = normal mice wound treated with HA MGs, NM (EXP.) = normal mice wound treated with HA‐LA granular gel, DM (CON.) = diabetic mice wound treated with HA MGs, DM (EXP.) = diabetic mice wound treated with HA‐LA granular gel) (scale bar: 50 μm, n = 3). The data represent the mean ± SD. *P < .05, **P < .01, ***P < .001, according to *t*‐test. HA‐LA, hyaluronic acid‐*g*‐lipoic acid; HA MGs, hyaluronic acid microgels; ROS, reactive oxygen species; SD, standard deviation

It is well known that the integrin α‐3 can be used as a marker for tissue regeneration and collagen.^37^ Therefore, immunofluorescence staining of wound tissue with integrin α‐3 can demonstrate tissue regeneration directly. In addition, CD31 is a marker of neovascularization,^38^ and α‐SMA is a specific marker of myofibroblasts,^39^ which play essential roles in wound contraction and tissue fibrosis. These three markers can also serve as indicators of tissue microenvironment repair. Therefore, CD31, integrin α‐3, and α‐SMA immunofluorescence staining were performed on the skin wound tissue. Figure 9a‐i shows that the levels of three indicators in the NM (EXP.) group increased significantly on day 7, indicating that the increase in wound neovascularization and collagen production was the most obvious after HA‐LA granular gel treatment. The significant increase of α‐SMA suggested that the NM (EXP.) group entered the proliferation stage earlier than other groups. On day 14, the expression of the three indicators decreased significantly in the NM (EXP.) group while increasing significantly in the DM (BLANK) and DM (CON.) groups, indicating that the NM (EXP.) group had passed the proliferation stage and progressed to the remodeling stage. In contrast, the DM (BLANK) and DM (CON.) groups began to show significant neovascularization and collagen generation, which was in the proliferation stage and delayed tissue repair. The fluorescence intensity of three indexes of the DM (EXP.) group was lower than that of the DM (BLANK) and DM (CON.) groups, indicating that the healing was better than the DM (BLANK) and DM (CON.) groups, but not as good as the NM (EXP.) group.

Compared with nondiabetic patients, because wounds in diabetic patients are more prone to long‐term hyperglycemia and bacterial infection, this can increase the number of neutrophils and macrophages, lead to an enhanced inflammatory response, increase ROS levels, and aggravate tissue damage. Research has shown that the microenvironment of excessive ROS and bacterial infection can promote the expression of M1 macrophage markers while decreasing the expression of M2 macrophage markers.^40^ Decreasing ROS levels can partially reverse the M1/M2 macrophage polarization, inhibit M1 macrophages, and restore M2 macrophages.^41^ Additionally, M2 phenotype macrophages can promote angiogenesis and collagen production, thus playing important roles in wound healing.^42^, ^43^ In the present study, excessive ROS was eliminated after applying HA‐LA granular gel to the wound surface. The polarization of macrophages might be directed from the M1 to M2 type, reducing the inflammatory response and promoting tissue regeneration. In conclusion, the HA‐LA granular gel can eliminate excessive ROS and effectively promote wound healing by increasing the levels of tissue regeneration and neovascularization markers such as CD31, integrin α‐3, and α‐SMA.

### 3.6 RNA‐seq analysis in the microenvironment during wound healing

**FIGURE 10:**
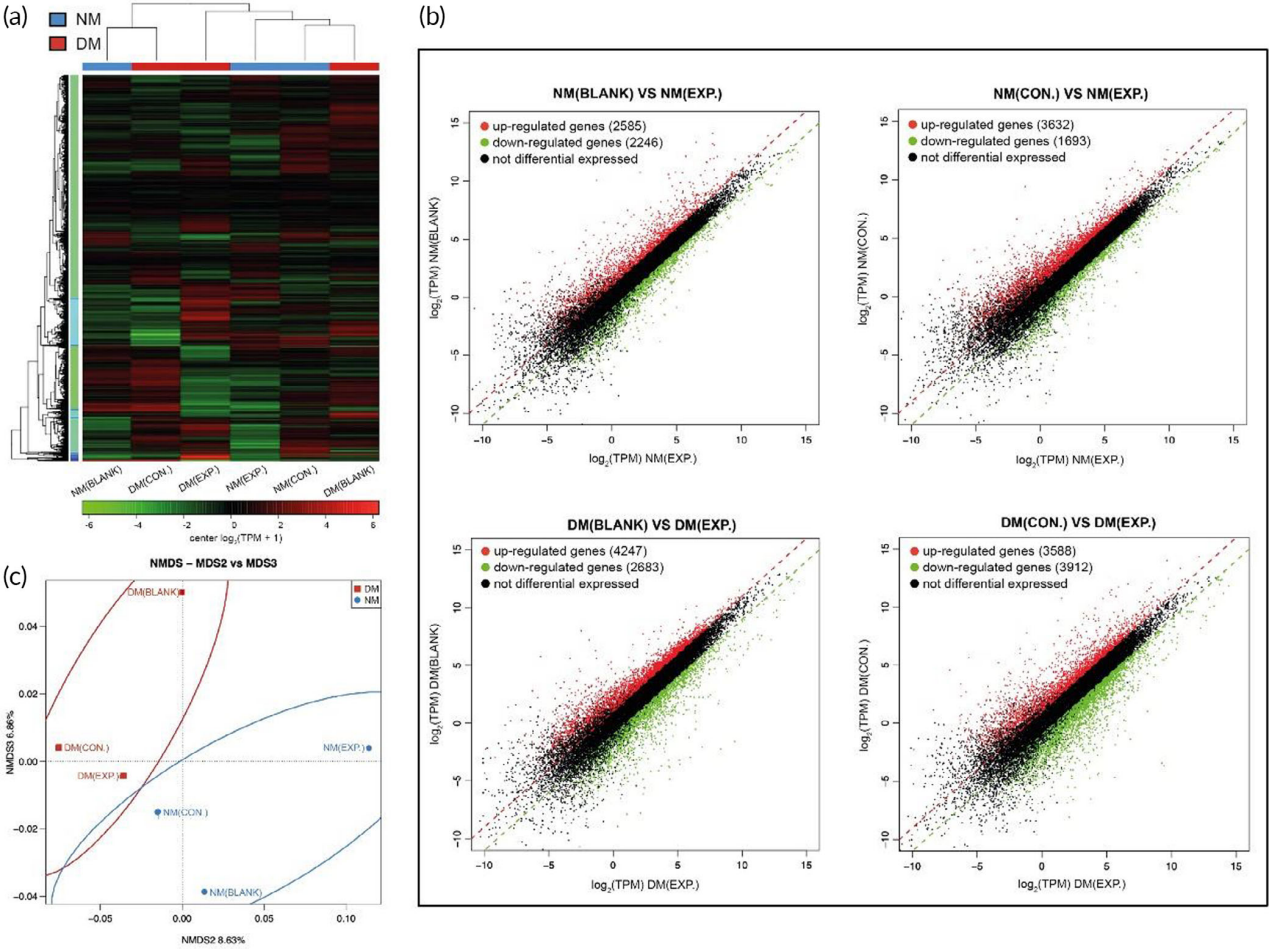
Transcriptomics data of wound tissue in all groups on day 7. (A) Heatmap showed the differential expression of RNA sequencing data from the wound tissue in all groups. (B) Scatter map showed the differential expression of RNA sequencing data from the wound tissue in all groups. (C) NMDS analysis showed the differential expression of RNA sequencing data from the wound tissue in all groups (NM (CON.) = normal mice wound treated with HA MGs, NM (EXP.) = normal mice wound treated with HA‐LA granular gel, DM (CON.) = diabetic mice wound treated with HA MGs, DM (EXP.) = diabetic mice wound treated with HA‐LA granular gel, n = 3). HA‐LA, hyaluronic acid‐*g*‐lipoic acid; HA MGs, hyaluronic acid microgels; NMDS, nonmetric multidimensional scaling

To further clarify the effect of the HA‐LA granular gel in wound healing, we collected three pieces of day 7 skin wound tissues and performed RNA sequencing in groups of normal and diabetic mice. The results showed that many genes were differentially expressed in all groups on day 7. The pro‐inflammatory cytokine‐related TNF, IL1B, and IL‐6 genes were down‐regulated in the EXP. groups, while the M2‐type macrophage‐related MRC1, anti‐inflammatory cytokine‐related IL‐10, angiogenesis‐related VEGFA, PECAM1, and collagen‐related ITGA3 genes were up‐regulated (Figure 10a,b). Nonmetric multidimensional scaling (NMDS) showed that normal and diabetic mice gene expression had significantly different characteristics. The results demonstrated that the normal and diabetic mice group was highly clustered separately. Both NMDS2 (8.63%) and NMDS3 (6.86%) could significantly distinguish the two groups (Figure 10c).

**FIGURE 11:**
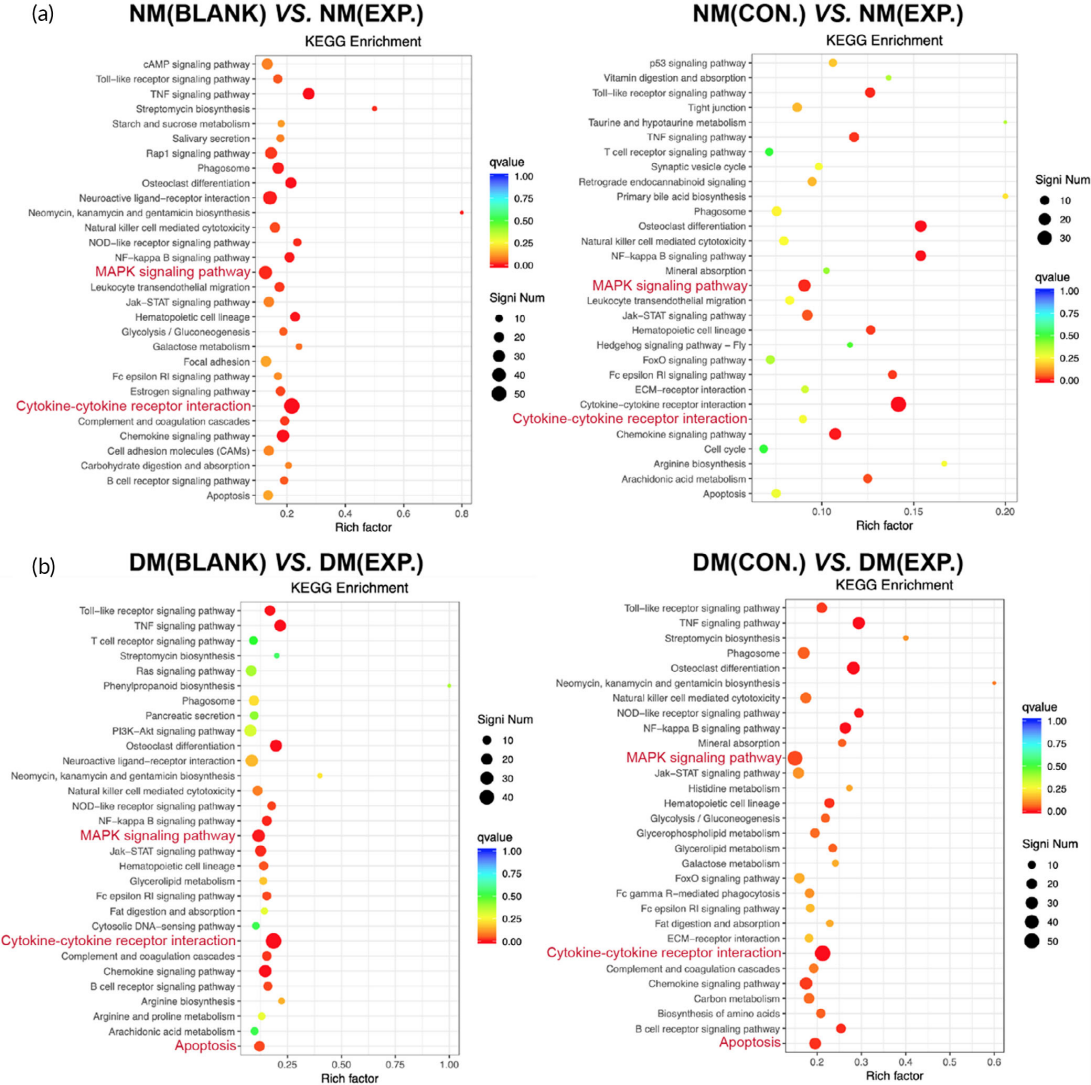
Transcriptomics data of wound tissue in all groups on day 7. (a and b) KEGG analysis of differentially expressed genes from the wound tissue in all groups (NM (CON.) = normal mice wound treated with HA MGs, NM (EXP.) = normal mice wound treated with HA‐LA granular gel, DM (CON.) = diabetic mice wound treated with HA MGs, DM (EXP.) = diabetic mice wound treated with HA‐LA granular gel, n = 3). HA‐LA, hyaluronic acid‐*g*‐lipoic acid; HA MGs, hyaluronic acid microgels; KEGG, Kyoto Encyclopedia of Genes and Genomes

The trend of different genes in each group was further analyzed through Kyoto Encyclopedia of Genes and Genomes (KEGG) analysis. The MAPK signaling pathway is activated after being stimulated and transmits extracellular signals through the cell membrane to the nucleus through phosphorylation and participates in various cellular functions such as cell proliferation, differentiation, and migration.^44^, ^45^ Growth factors, cytokines, and various stresses can activate ROS.^46^ Studies have shown that excess ROS induces oxidative modification of MAPK signaling proteins, such as MAP3K, thereby activating the MAPK signaling pathway.^47^ One of the branches of this pathway, ERK, is involved in the cellular inflammatory response.^48^ KEGG analysis showed that in the MAPK signaling pathway, IL1B, TNF, RASA2, MAP2K7, IKBKG, Nfkbia, CD14, and JUN genes were down‐regulated in the EXP. group of normal and diabetic mice compared with the BLANK group and CON. group. There was apparent gene enrichment, with the *q* value <0.05. Another point of concern is the cytokine‐cytokine receptor interaction. Cytokines regulate the inflammatory response, cell growth, cell differentiation, apoptosis, and angiogenesis on the cell surface of target cells.^49^ Furthermore, pancreatic β cells mediate oxidative and nitrosative stress responses in response to various stimuli, promoting the expression levels of IFN‐γ, IL‐1β, TNF‐α, IL‐1, and IL‐6. This results in the activation of transcription factors and nuclear factor‐κB in the extrinsic pathway of β cells, leading to apoptosis.^50^, ^51^ Therefore, in the KEGG analysis, the cytokine‐cytokine receptor interaction, there was apparent gene enrichment with the *q* value <0.05 and the *q* value of apoptosis signal in the DM group was lower than that in the NM group (Figure 11a,b). Overall, sequencing results indicated that the HA‐LA granular gel could reduce the release of pro‐inflammatory cytokines and promote wound healing through its ability to scavenge ROS by affecting the MAPK signaling pathway and cytokine receptor binding.

## 4 CONCLUSION

A multifunctional medical dressing was designed for diabetic wound surfaces. HA‐LA was synthesized and used to fabricate the microgels. HA‐LA MGs were assembled by Ag^+^ via its coordination effect with disulfide in dithiolane to form an injectable granular gel. The presence of LA and Ag^+^ endowed the gel dressing with ROS‐scavenging and antibacterial properties. The granular gel could be easily spread onto the wound and eliminate excessive ROS, which accelerated wound healing by increasing the levels of tissue regeneration and neovascularization markers such as CD31, integrin α‐3, and α‐SMA. Therefore, the ROS‐scavenging granular gel introduced in the present study could effectively regenerate wounds under various complex conditions and was expected to be used to treat difficult wounds, including diabetic infection.

## AUTHOR CONTRIBUTIONS

**Shixi Zhang:** Data curation (supporting); formal analysis (supporting); investigation (supporting); methodology (supporting); visualization (supporting); writing – original draft (lead); writing – review and editing (lead). **Yuqing Pan:** Formal analysis (supporting); investigation (supporting); methodology (supporting); visualization (supporting). **Zhiyuan Mao:** Investigation (supporting); methodology (supporting); visualization (supporting). **Jiahui Zhang:** Formal analysis (supporting); investigation (supporting); project administration (supporting); resources (lead); supervision (supporting); validation (supporting); visualization (supporting); writing – review and editing (supporting). **Kunxi Zhang:** Conceptualization (lead); funding acquisition (equal); project administration (supporting); supervision (lead); writing – original draft (lead); writing – review and editing (lead).

## FUNDING INFORMATION

This research was funded by National Natural Science Foundation of China (grant number 51973108, 201901047) and Natural Science Foundation of Shanghai (No. 22ZR1424700).

## CONFLICT OF INTERESTS

The authors declare no conflict of interest regarding the publication of this article.

### PEER REVIEW

The peer review history for this article is available at https://publons.com/publon/10.1002/btm2.10402.

## ETHICS STATEMENT

All experimental protocols in the study was conducted according to the guidelines of the Declaration of Helsinki and approved by the Institutional Ethics Committee of Shanghai Ninth People's Hospital, Shanghai Jiao Tong University School of Medicine (SH9H‐2021‐A32‐1). Informed consent was obtained from all subjects and/or their legal guardian(s). And all methods were carried out in accordance with relevant guidelines and regulations.

## ETHICS FOR USING MICE IN STUDY

Authors reporting experiments on live vertebrates must confirm that all experiments were approved by the Institutional Ethics Committee of Shanghai Ninth People's Hospital, Shanghai Jiao Tong University School of Medicine, and all experiments were performed in accordance with relevant guidelines and regulations. All methods are reported in accordance with ARRIVE guidelines (https://arriveguidelines.org) for the reporting of animal experiments.

## Supporting information


**Figure S1.** *M*_w_ of HA before and after lipoic acid conjugation tested by GPC
**Figure S2.** Microspheres were embedded in the newborn tissue in some H&E staining (scale bar = 100 μm)
**Table S1.** Synthesis of HA‐LA

## Data Availability

The data that support the findings of this study are available from the corresponding author upon reasonable request.
