# Supplementary material for: Hyaluronic acid‐g‐lipoic acid granular gel for promoting diabetic wound healing
Source: Bioeng Transl Med. 2022 Sep 14;8(2):e10402. doi: 10.1002/btm2.10402 (PMC10013829; doi:10.1002/btm2.10402)
Supplement: Supplementary file 1 — Figure S1. M w of HA before and after lipoic acid conjugation tested by GPC Figure S2. Microspheres were embedded in the newborn tissue in some H&E staining (scale bar = 100 μm) Table S1. Synthesis of HA‐LA [file BTM2-8-e10402-s001.docx]

**Supporting Information**

**Hyaluronic acid-*g*-lipoic acid granular gel for promoting diabetic wound healing**

Shixi Zhang ^a^, Yuqing Pan ^b^, Zhiyuan Mao ^a^, Jiahui Zhang ^b^, Kunxi Zhang ^b,^*, Jingbo Yin ^b,^*, Chen Wang ^a,^*

^a^ Department of Plastic and Reconstructive Surgery, Shanghai 9th People’s Hospital, Shanghai Jiao Tong University School of Medicine, Shanghai 200011, PR China.

^b^ Department of Polymer Materials, School of Materials Science and Engineering, Shanghai University, Shanghai 200444, PR China.

**Corresponding Author**

*E-mail: [zhangkunxi@shu.edu.cn](mailto:zhangkunxi@shu.edu.cn) (K. Zhang).

*E-mail: [jbyin@oa.shu.edu.cn](mailto:jbyin@oa.shu.edu.cn) (J. Yin).

*E-mail: [wangchen2369@163.com](mailto:wangchen2369@163.com) (C. Wang)


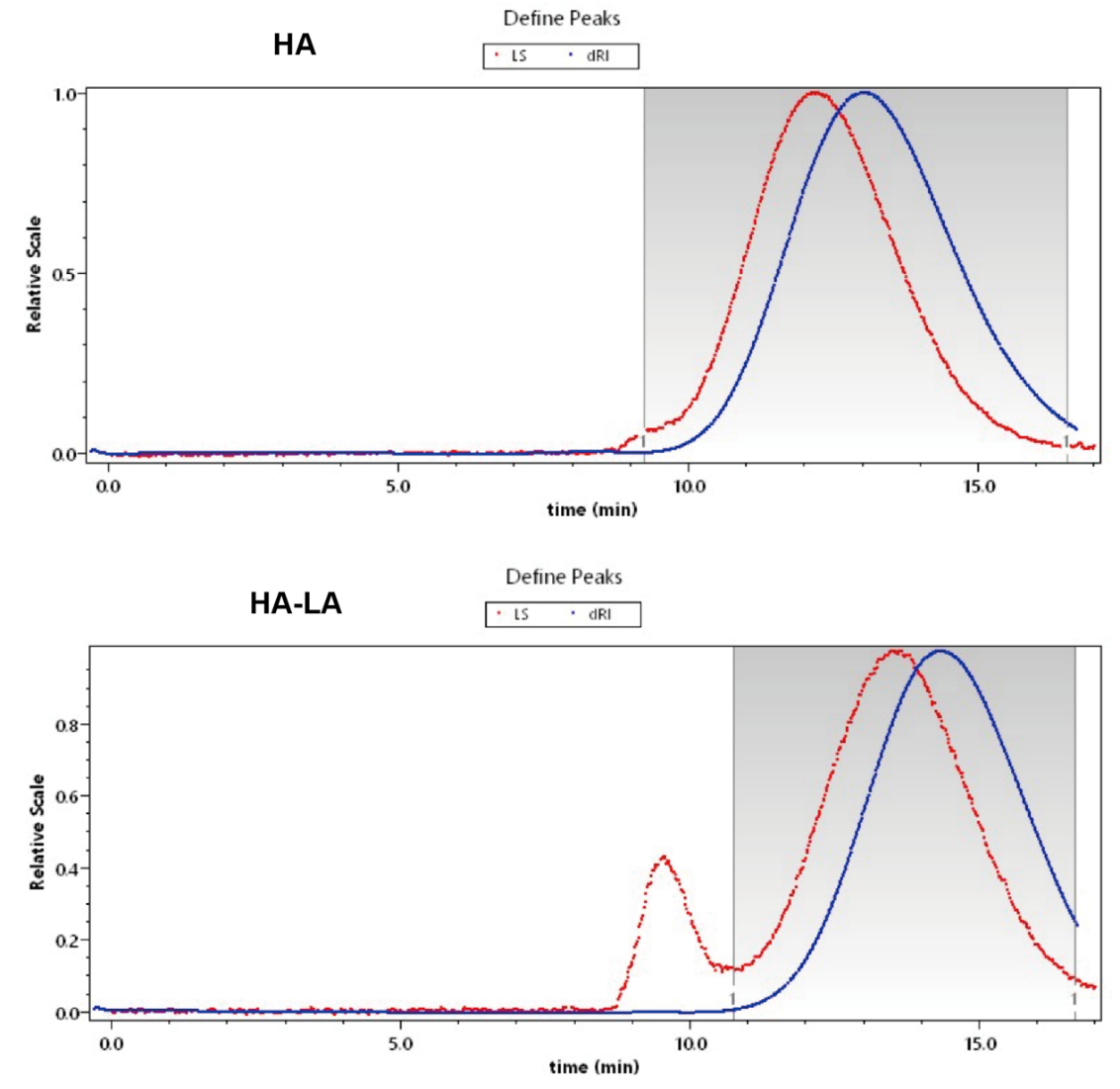


Fig. S1 M_w_ of HA before and after lipoic acid conjugation tested by GPC.


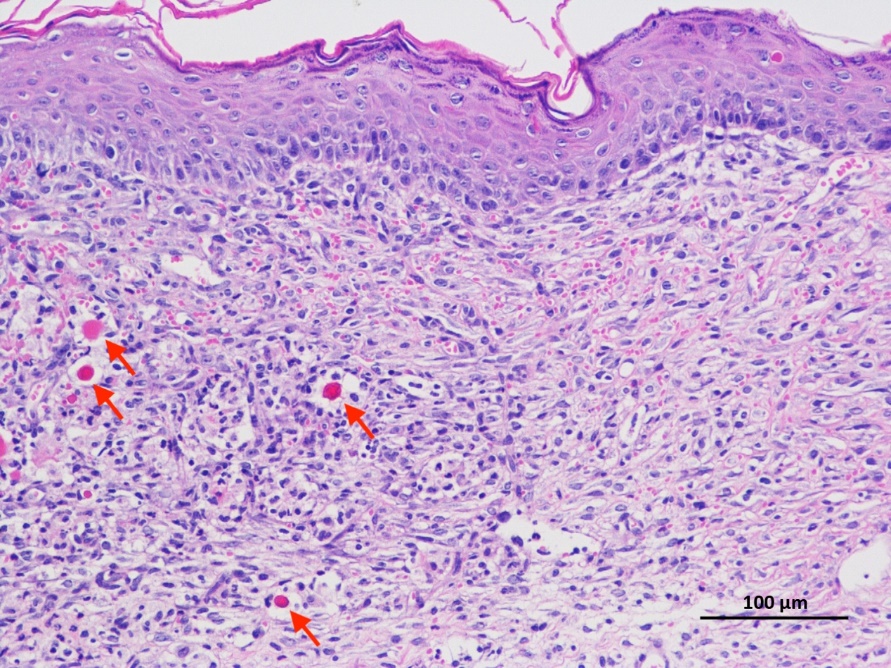


Fig. S2 Microspheres were embedded in the newborn tissue in some H&E staining (Bar scale = 100 μm).

Table. S1 Synthesis of HA-LA

|  | HA | HA-LA |
| --- | --- | --- |
| M_n_ (g/mol) | 3.037 × 10^4^ (±2.068%) | 1.672 × 10^4^ (±1.480%) |
| M_w_ (g/mol) | 4.458 × 10^4^ (±0.998%) | 2.311 × 10^4^ (±1.079%) |
| M_z_ (g/mol) | 8.177 × 10^4^ (±3.130%) | 3.927 × 10^4^ (±2.878%) |
| M_w_/M_n_ | 1.468 (±2.296%) | 1.382 (±1.832%) |
| M_z_/M_n_ | 2.693 (±3.752%) | 2.349 (±3.237%) |
